# Perinatal DDT Exposure Induces Hypertension and Cardiac Hypertrophy in Adult Mice

**DOI:** 10.1289/EHP164

**Published:** 2016-06-21

**Authors:** Michele A. La Merrill, Sunjay Sethi, Ludovic Benard, Erin Moshier, Borje Haraldsson, Christoph Buettner

**Affiliations:** 1 Department of Environmental Toxicology, University of California, Davis, Davis, CA, USA; 2 Department of Preventive Medicine, and; 3 Center for Science and Medicine, Icahn School of Medicine at Mount Sinai, New York, New York, USA; 4 Department of Nephrology, University of Gothenburg, Gothenburg, Sweden; 5 Department of Medicine,; 6 Department of Neuroscience, and; 7 Diabetes, Obesity and Metabolism Institute, Icahn School of Medicine at Mount Sinai, New York, New York, USA

## Abstract

**Background:**

Dichlorodiphenyltrichloroethane (DDT) was used extensively to control malaria, typhus, body lice, and bubonic plague worldwide, until countries began restricting its use in the 1970s. However, the use of DDT to control vector-borne diseases continues in developing countries. Prenatal DDT exposure is associated with elevated blood pressure in humans.

**Objective:**

We hypothesized that perinatal DDT exposure causes hypertension in adult mice.

**Methods:**

DDT was administered to C57BL/6J dams from gestational day 11.5 to postnatal day 5. Blood pressure (BP) and myocardial wall thickness were measured in male and female adult offspring. Adult mice were treated with an angiotensin converting enzyme (ACE) inhibitor, captopril, to evaluate sensitivity to amelioration of DDT-associated hypertension by ACE inhibition. We further assessed the influence of DDT exposure on the expression of mRNAs that regulate BP through renal ion transport.

**Results:**

Adult mice perinatally exposed to DDT exhibited chronically increased systolic BP, increased myocardial wall thickness, and elevated expression of mRNAs of several renal ion transporters. Captopril completely reversed hypertension in mice perinatally exposed to DDT.

**Conclusions:**

These data demonstrate that perinatal exposure to DDT causes hypertension and cardiac hypertrophy in adult offspring. A key mechanism underpinning this hypertension is an overactivated renin angiotensin system because ACE inhibition reverses the hypertension induced by perinatal DDT exposure.

**Citation:**

La Merrill M, Sethi S, Benard L, Moshier E, Haraldsson B, Buettner C. 2016. Perinatal DDT exposure induces hypertension and cardiac hypertrophy in adult mice. Environ Health Perspect 124:1722–1727; http://dx.doi.org/10.1289/EHP164

## Introduction

Cardiovascular disease is the number-one cause of overall mortality throughout the world (Yusuf et al. 2015), and hypertension is a major risk factor (Wilkins et al. 2012). Hypertension afflicts over a quarter of the world’s adults, with nearly twice as many cases of hypertension in the developing world than in the developed world (Kearney et al. 2005).

Most cases of hypertension are considered essential hypertension for which a clear cause is not clinically identifiable (Messerli et al. 2007). It may be postulated that some of these cases of hypertension arise from causes such as perturbations in fetal or early-life development. This hypothesis is supported by strong epidemiological and experimental evidence. For example, fetal malnutrition results in hypertension and cardiovascular disease in adulthood (Alwasel and Ashton 2009; Barker et al. 1989, 1993; Woods et al. 2001). Furthermore, perinatal administration of the glucocorticoid dexamethasone causes hypertension and an increase in the expression of renal ion transporters in adult rat offspring (Dagan et al. 2008). These observations support the paradigm that the nutritional environment of the fetus during critical developmental periods may lead to impaired blood pressure control in adulthood.

There is some evidence that perinatal exposure to environmental toxicants can cause cardiovascular disease in adulthood. For example, prenatal exposure to the pesticide dichlorodiphenyltrichloroethane (DDT) is associated with increased medicated hypertension in adult women (La Merrill et al. 2013), and prenatal exposure to its metabolite dichlorodiphenyldichloroethylene (DDE) is associated with elevated blood pressure in 4-year-old children (Vafeiadi et al. 2015). However, whether the DDT or DDE burden in *adults* is associated with hypertension remains somewhat controversial because some studies do (Henríquez-Hernández et al. 2014; Lind et al. 2014; Siddiqui et al. 2002), and some studies do not, report an association between DDT burden and hypertension in adult offspring (Goncharov et al. 2011; Savitz et al. 2014; Valera et al. 2013).

This discrepancy led us to hypothesize that perinatal exposure to DDT is a key risk factor for hypertension in adults. The present study sought to experimentally test the hypothesis that developmental exposure to DDT causes hypertension in adult offspring of mice.

Given the continued use of DDT in developing nations, and given the widespread presence of its metabolite DDE in particular, even a modest effect of DDT or its metabolites on blood pressure or cardiovascular disease may have far-reaching public health implications.

## Methods

### Drugs

*p*,*p*´-DDT (98.5% purity neat) and *o*,*p*´-DDT (100% purity neat) were purchased from AccuStandard (New Haven, CT). To simulate the relative abundance of these congeners in the commercial formulation of DDT that was used as a pesticide in the United States before its ban, 77.2% *p*,*p*´-DDT and 22.8% *o*,*p*´-DDT were dissolved in organic olive oil at a final concentration of 0.17 g DDT mixture/L (Ecobichon and Saschenbrecker 1968), hereafter referred to as DDT. Captopril (98% purity) was purchased from Sigma-Aldrich (St. Louis, MO). Captopril was dissolved at concentrations of 0.49 mg/mL and 0.57 mg/mL in the drinking water of female and male mice, respectively, based on the water intake of two co-housed male or female mice that had prior exposure to the DDT dose used here or to its control (see Table S1).

***Mice.*** Virgin 8-week-old C57BL/6J male and female mice were ordered from Jackson Laboratories and were acclimated for 1–2 weeks before timed mating.

***DDT exposure and mouse husbandry.*** We administered 1.7 mg DDT/kg body weight [*per os* (*p*. *o*.), *n* = 15 dams, 10 μL solution/kg body weight], or the equivalent volume of olive oil vehicle (hereafter referred to as vehicle; *p*. *o*., *n* = 14 dams) daily to primigravid C57BL/6J dams from 11.5 days post coitus (DPC) to postnatal day (PND) 5 to span a developmental exposure window important for rodent kidney and heart function (Aragon et al. 2008; Couture et al. 1990; Lin et al. 2001; Thackaberry et al. 2005; Xu et al. 2011). We previously reported mean (± SEM) maternal serum levels (in nanograms/milliliter serum) of 2.2 (± 0.1) *p*,*p*´-DDE, 51.1 (± 10.2) *p*,*p*´-DDT, and no detectable *o*,*p*´-DDT on PND 6, 24 hr after an identical daily dosing protocol of 1.7 mg/kg from 11.5 DPC to PND5 (La Merrill et al. 2014). These exposures are within the range of past and contemporary human serum levels of both *p*,*p*´-DDT and *p*,*p*´-DDE (Bouwman et al. 1992; Cox et al. 2007; Gauthier et al. 2014; Herrera-Portugal et al. 2005; La Merrill et al. 2013; Rylander et al. 2009; Vafeiadi et al. 2015).

After the final DDT dose on PND 5, we culled litters to six pups to equalize litter size. At PND 21, we weaned pups (one cage/sex/litter/treatment) for later experiments. All mice had access to food and water *ad libitum* in sterile ventilated cages in an American Association for the Accreditation of Laboratory Animal Care–approved facility on a 12/12 light/dark cycle corresponding to 0700/1900 hours. At the end of the studies, mice were sacrificed by exsanguination under isoflurane anesthesia. All procedures were in accordance with the University of California (UC), Davis and Mount Sinai School of Medicine Institutional Animal Care and Use Committee (IACUC) protocols.

### Blood Pressure Measurements and Angiotensin Converting Enzyme (ACE) Inhibitor Treatment

Based on our previous finding that prenatal DDT exposure increased risk of hypertension in adult women (La Merrill et al. 2013), we assessed the development of adult male and female mouse hypertension (Monassier et al. 2006) by measuring blood pressure with a volume pressure recording sensor and an occlusion cuff (CODA, Kent Scientific) on the tail. We recorded 18 volume pressure cycles per restrained mouse in two mice/sex/litter/perinatal treatment group when they were 5 months old after a day of acclimation to the procedure (see Table S2). We next tested whether an over-activated renin angiotensin system (RAS) could be a key contributor of DDT effects by treating 7-month-old (adult) male and female mice with an angiotensin converting enzyme inhibitor (ACEi) and measuring blood pressure using two independent methods in the ACEi studies (see Table S2).

We recorded 18 volume pressure cycles per tail-cuffed and restrained mouse in two male and two female mice for seven litters/perinatal treatment group before the initiation of captopril treatment (ACEi group) or untreated water (water group). The recording procedure was repeated both 6 and 7 days after the initiation of 7 days of treatment with ACEi or water to assure sufficient time for pharmacological efficacy (Emanueli et al. 1997). Mice were acclimated to the tail-cuff procedure for a day prior to the measurements. Daily water intake was quantified before captopril treatment to appropriately target a daily intake of ~120 mg captopril/kg body weight (Emanueli et al. 1997). Daily water intake during 7 days of daily captopril treatment indicated average daily doses of 120 and 109 mg captopril/kg in female and male mice, respectively (see Table S1).

To validate these noninvasive blood pressure measurements conducted on restrained mice, we assessed blood pressure using an indwelling telemetric blood pressure device in a subgroup of unrestrained male mice (see Table S2; PA-C10, DSI, St. Paul, MI). Transmitters were implanted into the aortic arch of six male mice/perinatal treatment during isoflurane anesthesia. Mice recovered from surgery for 5 days, receiving analgesic buprenorphine twice daily for 3 days following surgery. Blood pressure was assessed in 7-month-old male mice by telemetry for 1 hr daily from 1700 to 1800 hours for 5 days (water group) and from days 7 to 12 (ACEi group). We added captopril to the drinking water on the fifth day (see Table S1). Because of some postsurgical mortality and device malfunctions, not all males that underwent surgery completed the ACEi study, resulting in six males in the vehicle+water treatment group, four males in the DDT+water treatment group, three males in the vehicle+ACEi treatment group and four males in the DDT+ACEi treatment group.

While 7-month-old male mice were subjected to telemetric blood pressure measurements, their age-matched sisters were subjected to an additional volume pressure recording of 18 volume pressure cycles per tail-cuffed and restrained mouse (one female mouse/litter and six litters/treatment; see Table S2) prior to euthanasia. This procedure was performed to confirm that the perinatal treatment effect was present at the time of transcript analysis (see “Semi-quantitative PCR” below).

***Cardiac echography.*** Because we hypothesized that subtle increases in blood pressure resulting from perinatal DDT exposure could lead to cardiac hypertrophy, we evaluated cardiac phenotypes of male and female mice by echocardiography a month after significantly elevated blood pressure was observed in both sexes (see Table S2). Echocardiography was performed on 8-month-old mice (1 mouse/sex/litter, 7 litters/treatment, 14 mice/treatment) under sedation by intraperitoneal ketamine ≤ 75 mg/kg. Sedation was optimized by giving the lowest dose of ketamine needed to *a*) restrain the animal and prevent motion artifact and *b*) maintain the heart rate in the range of 550–650 beats/minute. Ketamine was chosen based on our previous experience and considering that alternative agents had either a long duration of action (pentobarbital), were potentially unsafe because of increased cardiac toxicity, or might cause a bradycardic effect (isoflurane, ketamine/xylazine) as demonstrated elsewhere (Stein et al. 2007). Short-axis parasternal views of the left ventricle (LV) at the mid-papillary level were obtained using a Vivid i echocardiography apparatus with a 13 MHz linear array probe (General Electric, New York, NY) on mice with hair removed (Nair™, Church & Dwight Co., Inc.). M-mode measurements of the size of the LV walls and cavities were obtained by two-dimensional (2D) guidance from the short-axis view of the LV as recommended by the American Society of Echocardiography (Lang et al. 2005). Three different measurements in diastole (d) were averaged per animal to estimate LV wall thicknesses (septum and posterior wall) and LV dilation (internal diameter).

***Pathology.*** After 8-month-old mice had completed echography, kidneys were harvested from one mouse/sex/perinatal treatment and subjected to routine hematoxylin and eosin processing (see Table S2). Histopathological evaluations were assessed by a veterinary pathologist who was blinded to the treatments.

***Semiquantitative polymerase chain reaction.*** Renal mRNA was extracted (Qiagen, Hilden, Germany) from 7-month-old female mice to synthesize cDNA using reverse transcription polymerase chain reaction (PCR) (Applied Biosystems, Foster City, CA). Semiquantitative PCR was performed using SYBR® Green probes (Applied Biosystems) with primers for transcripts: sodium hydrogen exchanger 1 [*Slc9a1*, forward (F): CCT​GAC​CTG​GTT​CAT​CAA​CA, reverse (R): TCA​TGC​CCT​GCA​CAA​AGA​CG (Stiernet et al. 2007)]; sodium hydrogen exchanger 2 [*Slc9a2*, F: TGG​CAG​AGA​CAG​GGA​TGA​TAA​G, R: CCG​CTG​ACG​GAT​TTG​ATA​GAG​ATT​C (Stiernet et al. 2007)]; sodium hydrogen exchanger 3 [*Slc9a3*, F: GCA​CAC​AAC​TAC​ACC​ATC​AAG​G, R: AGG​GGA​GAA​CAC​GGG​ATT​ATC (Stiernet et al. 2007)]; sodium hydrogen exchanger 4 [*Slc9a4*, F: CGG​AGG​AAC​CTG​CCA​AAA​TC, R: CGG​AGG​AAC​CTG​CCA​AAA​TC (Stiernet et al. 2007)]; sodium phosphate transporter [*Slc34a1*, F: GCT​GTC​CTC​TAC​CTG​CTC​GTG​TG, R: GCG​TGC​CCA​CTC​CGA​CCA​TAG (Marsell et al. 2008)]; sodium potassium ATPase subunit 1 [*Atp1a1*, F: CGG​AAT​TCA​TGC​GGA​GGA​TGT​CGTC, R: GCC​GCT​CGA​GGT​GGA​TGA​AAT​GCT​CAA​T (Klesert et al. 2000)]; sodium potassium ATPase subunit 2 [*Atp1a2*, F: GAA​TGG​GTT​TCT​ACC​ATC​GCG, R: GCA​CAG​AAC​CAC​CAC​GTG​AC (Marsell et al. 2008)]; sodium calcium exchanger 1 [*Slc8a1,* F: TGA​GAG​GGA​CCA​AGA​TGA​TGA​GGA​A, R: TGA​CCC​AAG​ACA​AGC​AAT​TGA​AGA​A (Otsu et al. 2005)]; sodium potassium chloride cotransporter 2 [*Slc12a2*, F: GAA​CCT​TTT​GAG​GAT​GGC, R: CAC​GAT​CCA​TGA​CAA​TCT (Castrop et al. 2005)]; sodium potassium chloride cotransporter 1 [*Slc12a1*, F: TGC​TAA​TGG​AGA​TGG​GAT​GC, R: CAG​GAG​AGG​GCA​ATG​AAG​AG (Alshahrani et al. 2012)]. The 2^–ΔΔCT^ method (Livak and Schmittgen 2001) was used with 18s [*s18,* F: TTG​ACG​GAA​GGG​CAC​CAC​CAG, R: GCA​CCA​CCA​CCC​ACG​GAA​TCG (Au et al. 2011)] as an endogenous control in kidneys to calculate transcript fold change.

***Statistical analyses.*** The normal distribution was evaluated for all outcome variables here. Tail blood pressure was assessed by modeling the fixed effect of perinatal DDT and the random effect of litter (PROC MIXED; SAS 9.4, SAS Institute Inc., Cary, NC). In the ACEi study, ACEi and an ACEi * perinatal treatment term were modeled as fixed effects in the tail blood pressure model. Models of arterial blood pressure measured by telemetry included the random effect of litter using PROC GLIMMIX, a model that does not require a normal outcome distribution. mRNA expression was evaluated without random effects because only one mouse per litter was analyzed (PROC GLM, SAS). We stratified by sex in all outcomes for which both sexes were evaluated.

## Results

### Perinatal DDT Elevates Blood Pressure in Adult Offspring

**Figure 1:**
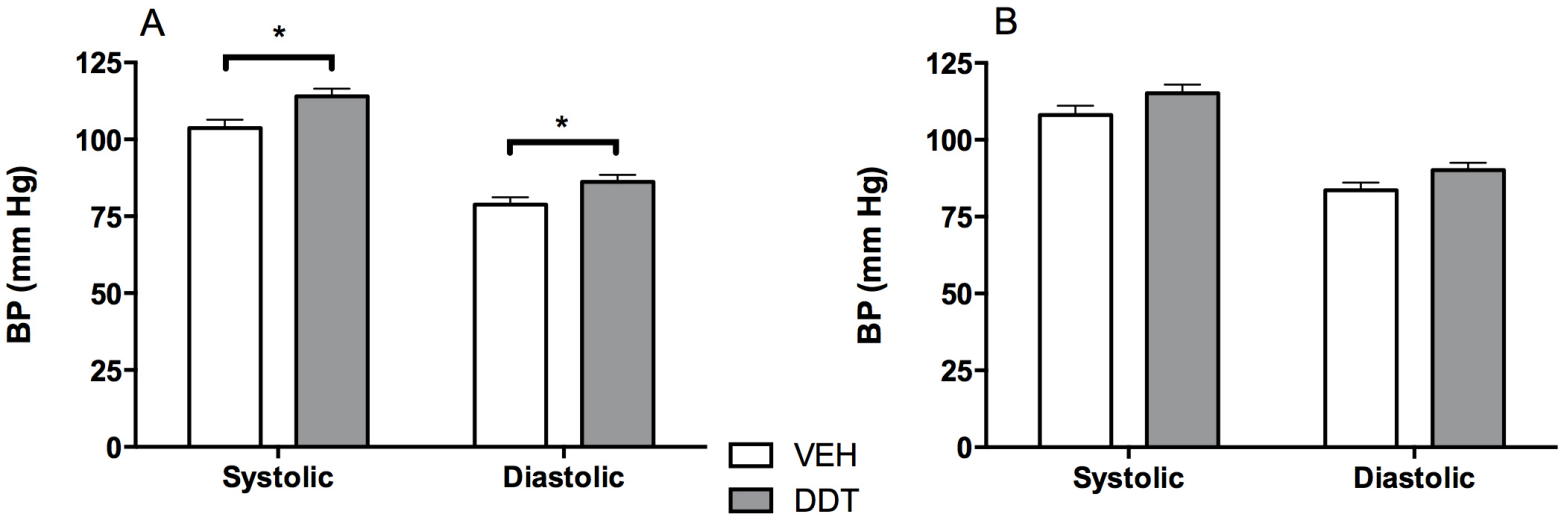
Perinatal DDT exposure increases blood pressure (BP) of 5-month-old male mice. Systolic and diastolic blood pressure (tail cuff) in 5-month-old (*A*) male and (*B*) female mice (DDT *p* = 0.1). *n* = 2 mice/sex/litter, 15 DDT litters, and 14 vehicle-exposed litters. *, *p *< 0.05, DDT vs. vehicle controls.

**Figure 2:**
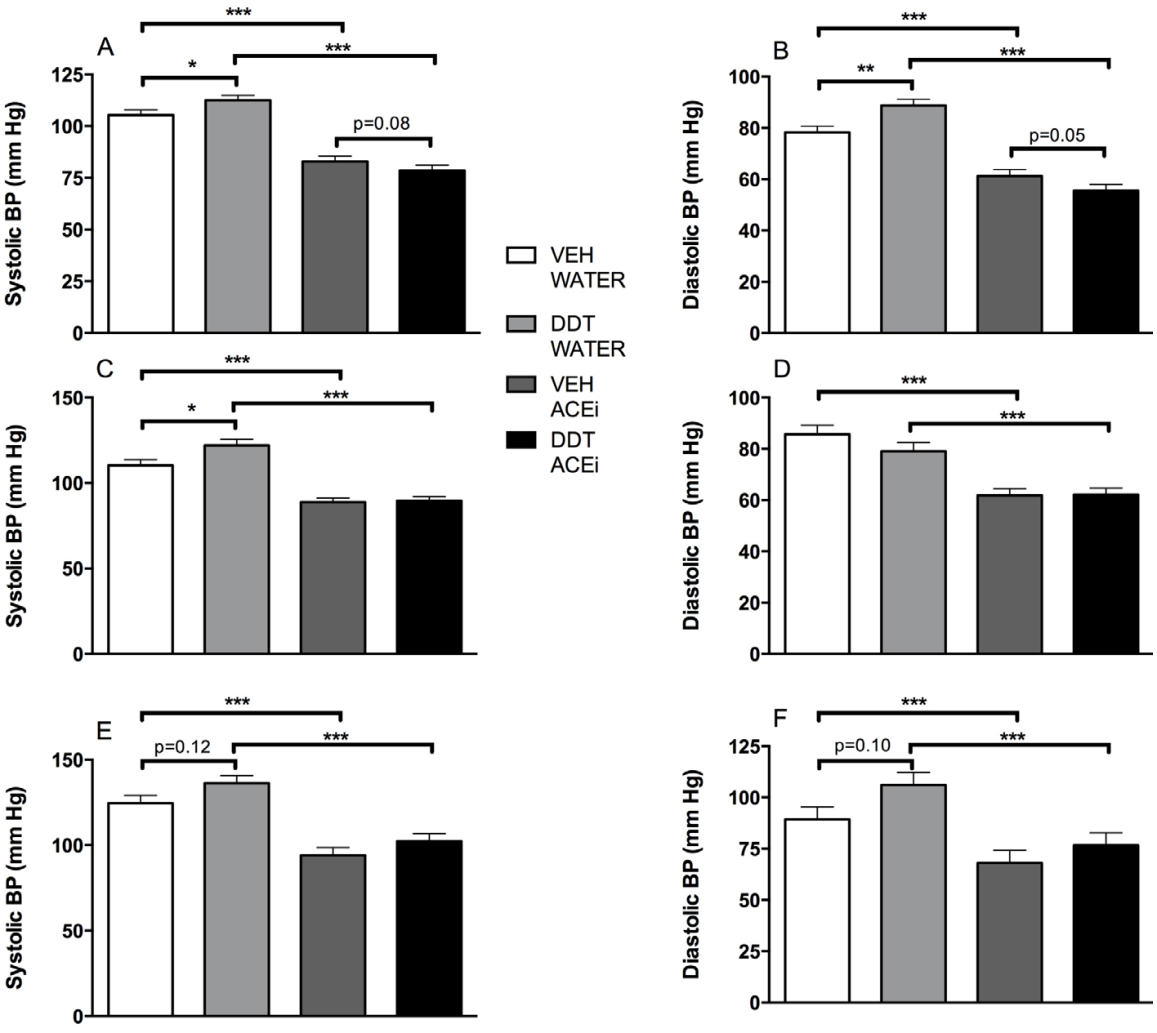
Increased blood pressure caused by perinatal DDT exposure is reduced by angiotensin converting enzyme inhibition (ACEi) in 7-month-old male and female mice. (*A*) Systolic (DDT*ACEi p_i_ = 0.02, *n* = 2 mice/litter, 7 litters/treatment) and (*B*) diastolic tail blood pressure (BP) (DDT*ACEi p_i_ = 0.002, *n* = 2 mice/litter, 7 litters/treatment) in female mice, and (*C*) systolic (DDT*ACEi p_i_ = 0.05, *n* = 2 mice/litter, 7 litters/treatment) and (*D*) diastolic (DDT*ACEi p_i_ = 0.19, *n* = 2 mice/litter, 7 litters/treatment) tail blood pressure in male mice at 7 months old immediately before and after 1 week of exposure to captopril in drinking water. (*E*) Systolic (DDT*ACEi p_i_ < 0.0001) and (*F*) diastolic (DDT*ACEi p_i_ < 0.0001) aortic blood pressure in 7-month-old male mice [*n* = 6, 4, 3, and 4 for vehicle (VEH)- and DDT-treated at baseline and VEH- and DDT-treated during captopril exposure, respectively]. *, *p *< 0.05; **, *p *< 0.01; ***, *p *< 0.0001.

**Figure 3:**
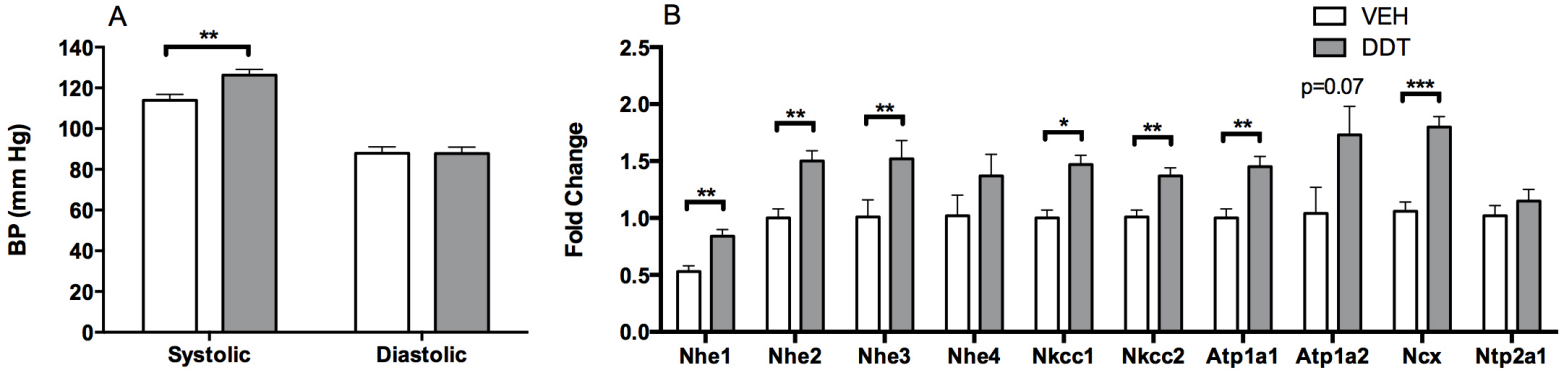
Perinatal DDT exposure increases blood pressure and renal sodium transporter expression in 7-month-old female mice. (*A*) Systolic and diastolic blood pressure (BP) immediately prior to the assessment of (*B*) renal sodium transporter transcripts in 7-month-old female mice that were not subjected to the angiotensin converting enzyme (ACE) inhibition experiment. *n* = 1 female mouse/litter and 6 litters/treatment. Atp1a1–2, sodium potassium ATPase subunit 1–2, Ncx, sodium calcium exchanger 1; Nhe1–4, sodium hydrogen exchanger 1–4; Nkcc1–2, sodium potassium chloride cotransporter 1–2; Ntp2a1, sodium phosphate transporter; VEH, vehicle. *, *p *< 0.05; **, *p *< 0.01; ***, *p *< 0.0001 DDT versus vehicle controls.

To assess whether the association between prenatal DDT exposure and blood pressure in adults is causal (La Merrill et al. 2013), we exposed fetuses and nursing pups to DDT by gavaging dams perinatally and measuring the blood pressure of adult offspring at 5 and 7 months of age. Blood pressure was assessed using two methods: noninvasively through tail-cuff blood pressure monitoring of restrained mice (Figures 1–3) and invasively through telemetric blood pressure monitoring (Figure 2E,F).

Male offspring that were perinatally exposed to DDT had elevated systolic and diastolic blood pressure at 5 months of age (Figure 1A). A similar but statistically nonsignificant trend was observed in 5-month-old female offspring (Figure 1B). By 7 months of age, both male and female offspring that had been exposed perinatally to DDT had increased systolic blood pressure (*p* < 0.05, Figures 2A,C and 3A).

To evaluate whether perinatal DDT increased blood pressure by increasing the activity of the RAS (Woods et al. 2001), we administered the ACEi captopril for 5–7 days to adult offspring in the study. Indeed, ACE inhibition lowered blood pressure in 7-month-old mice exposed to DDT to a greater extent than observed in the vehicle group (Figure 2; see Table S3), demonstrating that a primary cause of hypertension (Monassier et al. 2006) induced by perinatal DDT exposure is an over-activated RAS.

The well-known role of aldosterone in inducing sodium transport to elevate blood pressure (Rozansky 2006) led us to hypothesize that an over-activated RAS led to increased blood pressure via increased sodium transporter transcription in DDT-exposed mice. We tested this hypothesis by evaluating the expression of sodium transporter mRNA in female mice when they were 7 months old, the earliest time point at which we could detect a significant elevation in the blood pressure of female mice perinatally exposed to DDT (Figure 3A). Indeed, perinatal DDT exposure induced the mRNA expression of several Na^+^/H^+^ exchangers, two Na^+^/K^+^/Cl^–^ cotransporters, a subunit of the Na^+^/K^+^ ATPase, and a Na^+^/Ca^2+^ exchanger in hypertensive kidneys (Figure 3B), consistent with elevated expression of renal ion transporters in hypertension caused by an over-activated RAS (Rozansky 2006).

To rule out that DDT causes hypertension by impairing nephrogenesis (Langley-Evans et al. 1999), kidneys collected from 8-month-old male and female mice were examined by a veterinary pathologist. Histological assessment of the kidneys was completely normal at the light microscopy level (see Figure S1), supporting the notion that the cause of the hypertension induced by DDT is not structural in nature.

### Perinatal DDT Exposure Increases Heart Wall Thickness in Adult Offspring

**Figure 4:**
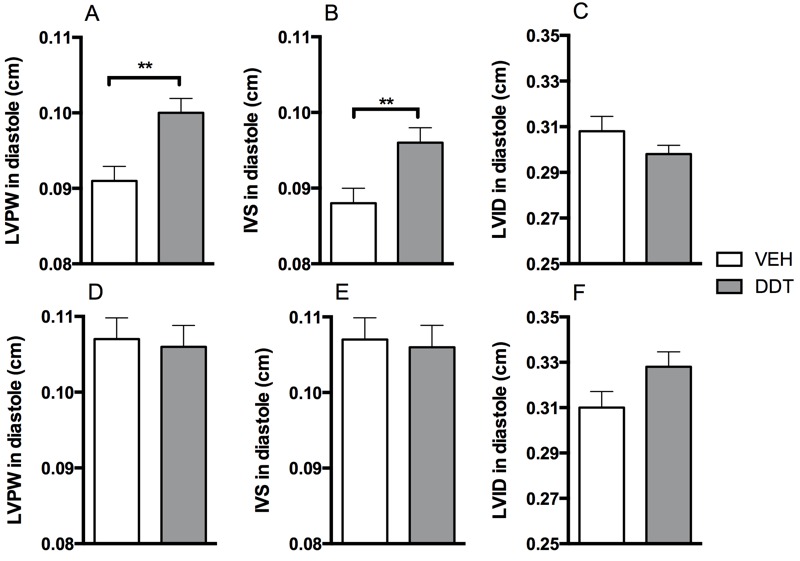
Perinatal DDT causes cardiac hypertrophy in 8.5-month-old female mice. (*A*) Left ventricular posterior wall (LVPW) thickness, (*B*) left inter-ventricular septum (IVS) thickness, and (*C*) left ventricular dilation (LVID) in diastole in 8-month-old female mice. (*D*) Left ventricular posterior wall thickness, (*E*) Left ventricular septum thickness, and (*F*) left ventricular dilation in diastole of male mice. *n* = 1 mouse/sex/litter, 7 litters/treatment. VEH, vehicle. **, *p *< 0.01 DDT versus vehicle controls.

Chronic hypertension can induce left ventricular hypertrophy as a result of pressure overload (Krumholz et al. 1993). We used echocardiography to investigate this possibility in 8-month-old male and female mice (a month after the last blood pressure measurements). We found that perinatal DDT exposure led to a significant increase in left ventricular wall thickness of both the posterior wall and the septum in female mice (Figure 4A,B); these changes are expected with chronic pressure overload. These data are consistent with the observation that hypertension in women favors the development of concentric hypertrophy (Krumholz et al. 1993).

Despite the observation that the onset of hypertension in mice exposed to DDT was earlier in males than in females, we did not observe any difference in ventricular wall thickness (Figure 4D–F). Further, there was no evidence of volume overload as assessed by left ventricular dilation (Figure 4C,F).

## Discussion

Here, we investigated the effects of perinatal DDT exposure on cardiovascular health in adult offspring. To our knowledge, this is the first study to establish that perinatal DDT exposure is sufficient to cause hypertension and cardiac hypertrophy in adult offspring (Monassier et al. 2006). Remarkably, the increase in blood pressure caused by perinatal DDT exposure was normalized by treatment with an ACE inhibitor, captopril, demonstrating that an over-activated RAS is a key mechanism in perinatal DDT exposure–induced hypertension. In turn, over-activated RAS was associated with increased renal expression of sodium channels.

The maternal serum levels of DDT and DDE that were achieved in this study were similar to the range of DDT and DDE levels observed in human maternal serum levels, particularly those that were associated with high blood pressure during childhood and hypertension in adult offspring (La Merrill et al. 2013; Vafeiadi et al. 2015). Thus, perinatal DDT exposure could be an important contributor in some of the hypertension that is currently still classified as “essential.” Our study mechanistically underpins associative evidence in humans that the latent effects of perinatal DDT exposure can clinically manifest (La Merrill et al. 2013) decades after environmental and human exposure levels have dramatically declined (Ritter et al. 2009). This research therefore provides a salient example of the legacy of detrimental health effects from persistent organic pollutants.

Interestingly, captopril caused a greater reduction of blood pressure in mice perinatally exposed to DDT than in controls, consistent with an over-activated RAS as a primary cause of DDT-induced hypertension. An over-activated RAS may also explain the increased expression of several ion transporters in the DDT-exposed hypertensive adult offspring because the expression of these transporters is regulated by the RAS (Rozansky 2006). Future studies should evaluate the effects of ACE inhibition on altered renal sodium channel expression after perinatal DDT exposure to confirm that DDT effects on sodium channel expression are RAS-mediated.

We observed modest concentric hypertrophy in the hearts of female adult mice perinatally exposed to DDT, likely a result of the hypertension (Monassier et al. 2006). This sexual dimorphism of greater cardiac wall thickness in hypertensive females has also been observed in humans (with some contention), where hypertensive women are more likely to develop concentric (Krumholz et al. 1993) and severe (Topol et al. 1985) cardiac hypertrophy than hypertensive men. Given that cardiac hypertrophy is associated with a higher risk of cardiovascular mortality in women than in men (Levy et al. 1990), future studies will need to elucidate the mechanism of the sex-specific results here because inherent sex-associated differences in cardiac hypertrophy could account for some of the sexual dimorphism of hypertrophy in DDT-exposed mice (Krumholz et al. 1993; Topol et al. 1985). Furthermore, whether developmental DDT exposure is associated with cardiac hypertrophy in humans, and if so, whether this is purely because of hypertension is a critical outstanding question that will need to be addressed.

The strengths of this study include the measurements of blood pressure by two independent methods that obtained similar results. Each of these methods has particular strengths, such as no requirement for surgery when using the tail cuff or lack of restraint stress with the use of telemetry, thus reaffirming the independent measurements. The study is further strengthened by the assessment of cardiac hypertrophy by echocardiography. Our dosing paradigm resulted in perinatal maternal levels of DDT and DDE that fell within the range of the top tertile based on the only human study that has evaluated and found a positive association between prenatal DDT exposure and adult hypertension, which adds to the translational value of the study (La Merrill et al. 2013). A limitation of the study is that we did not have a control group in which we induced a similar DDT burden in adulthood and assessed the effects on hypertension because we cannot rule out that the hypertension in the adult offspring is due to the remaining DDT burden associated with its long half-life. The increase in blood pressure with age while the DDT burden decreased over time suggests that any DDT remaining in our mice at the time of blood pressure assessment was not a major driver of the hypertension that we observed. Finally, whether ACE inhibition attenuates the perinatal DDT exposure–related elevation in transcripts associated with renal ion transport that we observed here remains to be determined.

## Conclusions

Our data show that perinatal DDT exposure caused hypertension in both female and male adult mice caused by an over-activation of the RAS. This chronic over-activation of the RAS was associated with increased renal expression of sodium channels. Further, perinatal DDT exposure caused cardiac hypertrophy, possibly through pressure overload. These observations support the hypothesis that perinatal exposure to DDT is a risk factor for hypertension and cardiovascular disease in adult offspring.

On a positive note, our study provides evidence that ACE inhibition normalizes hypertension of mice perinatally exposed to DDT. ACE inhibitors are among the most commonly used antihypertensive drugs that have been demonstrated to be safe and to reduce mortality from heart disease (Li et al. 2014), indicating that they would be the drug of choice for the treatment of hypertensive individuals with high early-life DDT exposure. Given that the ban of DDT in the United States has reduced DDT exposure among the U.S. population (Ritter et al. 2009), the ongoing replacement of DDT with other malaria vector controls is likely to further decrease worldwide DDT exposures over time. Our study suggests that this change may reduce susceptibility to cardiovascular disease in future generations.

## Supplemental Material

(562 KB) PDF
