# Supplementary material for: Perinatal DDT Exposure Induces Hypertension and Cardiac Hypertrophy in Adult Mice
Source: Environ Health Perspect. 2016 Jun 21;124(11):1722–7. doi: 10.1289/EHP164 (PMC5089878; doi:10.1289/EHP164)
Supplement: (562 KB) PDF [file EHP164.s001.acco.pdf]

Environ Health Perspect
DOI: 10.1289/EHP164

Note to readers with disabilities: *EHP* strives to ensure that all journal content is accessible to all readers. However, some figures and Supplemental Material published in *EHP* articles may not conform to 508 standards due to the complexity of the information being presented. If you need assistance accessing journal content, please contact ehp508@niehs.nih.gov. Our staff will work with you to assess and meet your accessibility needs within 3 working days.

Supplemental Material

Perinatal DDT Exposure Induces Hypertension and Cardiac Hypertrophy in Adult Mice

Michele La Merrill, Sunjay Sethi, Ludovic Benard, Erin Moshier, Borje Haraldsson, and Christoph Buettner

Table of Contents

Table S1. Water- and captopril- intake during the 1 week period of captopril administration.
Table S2. Summary of ages and numbers of mice during experimental measurements.
Table S3. Decrease in systolic blood pressure (mmHg) by captopril ACE inhibition relative to water treated controls.
Figure S1. No renal pathology associated with perinatal DDT exposure. Kidney morphology as visualized by H&E stain looks normal in 8 month old mice. N = 1 mouse/sex/litter and 8 litters/treatment.

Table S1. Water- and captopril- intake during the 1 week period of captopril administration.

	Male		Female	
	Vehicle	DDT	Vehicle	DDT
Mean water intake, water ml/kg body weight/day (SE)	196 (8)	190 (8)	255 (6)	238 (6)*
Mean captopril intake, captropril mg/kg body weight, day (SE)	112 (5)	108 (5)	125 (3)	117 (3)

*p<0.05 DDT vs. vehicle controls within each sex

Table S2. Summary of ages and numbers of mice during experimental measurements. ACEi, angiotensin converting enzyme inhibitor (captopril); BP, blood pressure measured by CODA.

Age	Sex	Measurement	Sample Size (mice/litter)	Sample size (litter/treatment)
5	Male	BP	2	15 DDT,<br>14 VEHICLE
5	Female	BP	2	15 DDT,<br>14 VEHICLE
7	Male	BP, ACEi	2	7
7	Female	BP, ACEi	2	7
7	Male	telemetry	1	6 VEH+Water<br>4 DDT+Water<br>3 VEH+ACEi<br>4 DDT+ACEi
7	Female	BP, renal transporter RNA	1	6
8	Male	EchoMRI, renal pathology	1	8
8	Female	EchoMRI, renal pathology	1	8

Table S3. Decrease in systolic blood pressure (mmHg) by captopril ACE inhibition relative to water treated controls.

Method	Sex	Mean (SE) among Perinatal Vehicle	Mean (SE) among Perinatal DDT	Interaction p-value
Tail cuff	Female	22.6 (3.3)	33.9 (3.3)	0.02
Tail cuff	Male	21.5 (4.0)	32.7 (4.0)	0.05
Telemetry	Male	30.5 (0.5)	34.0 (0.3)	<0.0001

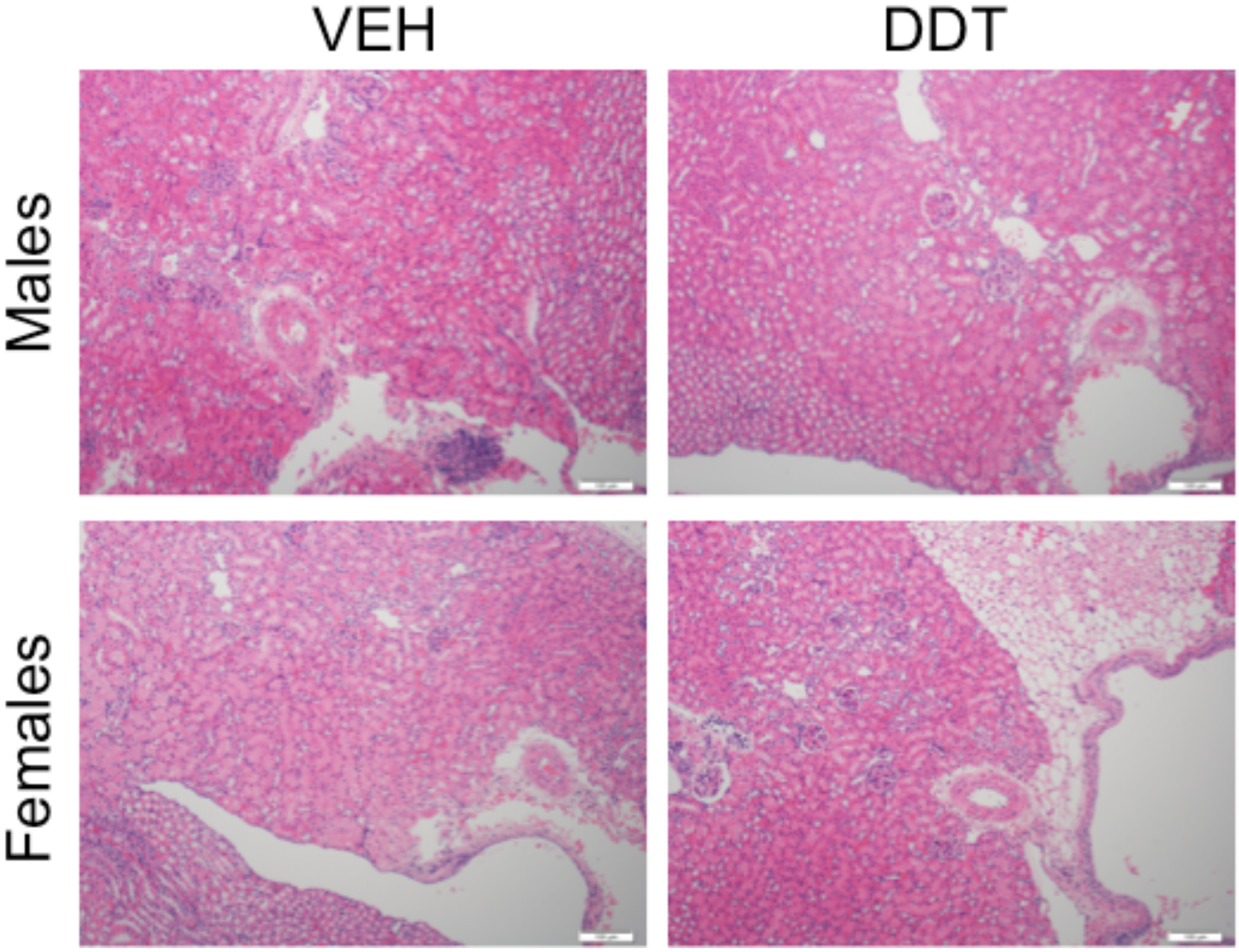

Figure S1. No renal pathology associated with perinatal DDT exposure. Kidney morphology as visualized by H&E stain looks normal in 8 month old mice. N = 1 mouse/sex/litter and 8 litters/treatment.
